# Recent Advancements of Magnetic Nanomaterials in Cancer Therapy

**DOI:** 10.3390/pharmaceutics12020147

**Published:** 2020-02-11

**Authors:** Sudip Mukherjee, Lily Liang, Omid Veiseh

**Affiliations:** Department of Bioengineering, George R. Brown School of Engineering, Rice University, Houston, TX 77005, USA; sudip.mukherjee@rice.edu (S.M.); ldl7@rice.edu (L.L.)

**Keywords:** magnetic nanoparticles (MNPs), cancer therapy, immunotherapy, toxicity, multifunctionality, theranostics

## Abstract

Magnetic nanomaterials belong to a class of highly-functionalizable tools for cancer therapy owing to their intrinsic magnetic properties and multifunctional design that provides a multimodal theranostics platform for cancer diagnosis, monitoring, and therapy. In this review article, we have provided an overview of the various applications of magnetic nanomaterials and recent advances in the development of these nanomaterials as cancer therapeutics. Moreover, the cancer targeting, potential toxicity, and degradability of these nanomaterials has been briefly addressed. Finally, the challenges for clinical translation and the future scope of magnetic nanoparticles in cancer therapy are discussed.

## 1. Introduction

Cancer is a disease of multiple etiology and described by unrestrained division of atypical cells in the body [1]. Despite major advancements over the past four decades aimed at improving the diagnosis and treatment cancer the disease still remains a global healthcare challenge [2]. Recent data by the American Cancer Society demonstrate that the global cancer burden will increase to 21.8 million new cases by the year 2030 [3]. The conventional treatment strategies including radiations, surgery, chemotherapy, photodynamic therapy alone or in combinations possess severe limitations that cause many side effects and toxicity issues. This has led an urgent need to design and develop an alternative therapeutic strategy in a targeted manner. In this viewpoint, nanomedicine is a revolutionary platform to prevailing over the existing challenges and develops a platform to combat cancer [4,5,6,7,8].

Magnetic nanoparticles (MNPs) have gained immense attention for cancer theranostics applications due to their unique physico-chemical properties, magnetic resonance imaging (MRI) contrast, facile synthesis, easy surface decorations, low toxicity, and good biodegradability that assist them to serve as outstanding imaging agents, and delivery vehicles in cancer theranostics [9,10,11,12,13,14,15,16,17,18,19,20,21,22,23]. MNPs act as capable (MRI) agents due to their increased magnetization upon application of an outer magnetic field along with excellent T2/T2* relaxation abilities [24,25,26]. Hence, MNPs are extensively utilized in various cancer theranostics applications including MRI imaging, biosensors, theranostics, delivery, magnetic hyperthermia, photodynamic therapy and photothermal ablation therapy [27,28,29,30,31,32,33]. Additionally, magnetic particle imaging (MPI) was drawing huge attention as an imaging tool using MNPs. The condition of the applied field, functionalization of magnetic nanoparticles, and particle structure for MPI was researched extensively by several research groups [12,13,14,15,16]. Importantly, USA Food and Drug Administration (FDA) has approved various MNPs based drugs including Feraheme®, Endorem®, Gastromark®, Lumiren®, Ferumoxytol®, Combidex®, Radiogardase®, and Feridex for various applications in iron deficiency, iron replacement therapy, the lymph node metastases imaging, MRI contrast agents or oral antidotes for heavy metal contamination in human [34,35,36]. Moreover, European Medicines Agency (EMA) lately approved NanoTherm® for the treatment of intermittent glioblastoma multiforme [37]. These examples obviously display the immense potential of MNPs in the applications of cancer therapy and diagnosis. 

In the present review, we have focused on to provide a detailed overview about the cancer therapeutic applications of MNPs including drug delivery, cancer immunotherapy, magnetic hyperthermia, photodynamic therapy, and anti-cancer agents. Moreover, the toxicity, pharmacokinetics, bio-distribution, and other challenges of MNPs in clinical translation have been briefly highlighted. However, we are not focusing on the imaging part including MPI of MNPs to keep our review article relevant to therapy. For more information on magnetic particle imaging (MPI) of MNPs the following articles can be referred [12,13,14,15,16]. Further, we encourage the authors to read the following review articles for additional information on cancer theranostics application of MNPs [17,18,19,20,21,22,23,24,25,26].

## 2. Synthesis, Characterization and Role of Size, Shape, and Surface Coating of MNPs in Cancer Therapy

Magnetic nanoparticles have been developed utilizing nickel, cobalt, Prussian blue, and gadolinium, but magnetic iron oxide (usually maghemite γ-Fe_2_O_3_ or magnetite Fe_3_O_4_) NPs remain the most extensively researched MNP-based cancer theranostics due to their low systemic toxicity and strong MRI contrast properties. MNPs generally consist of a magnetic core-shell and a polymer coating. The MNP surface coating and functionalization enhances colloidal stability, allows for covalent or electrostatic binding of therapeutic cargo, targeting moieties, and/or additional imaging probes, as well as play an important role in tuning MNPs properties such as pharmacokinetics, systemic toxicity and clearance rate, nonspecific protein adsorption or cell interactions, and sustained drug release, among others. Synthesis of MNPs traditionally includes co-precipitation of salts with stabilizing polymer, hydrothermal or solvothermal techniques, sonochemistry, reverse microemulsion, and thermal decomposition. Please refer to the subsequent reviews for more in-depth discussions of these synthesis techniques [38,39]. Recently, novel synthesis strategies have been developed such as microfluidic and biogenic synthesis. Microfluidic systems can utilize a wide range of materials such as glass, silicon, ceramic, polymers, and stainless steel to form geometrically constrained channels for nanoparticles synthesis. Microfluidic systems provide advantages such as process screening, automation, and continuous synthesis, increased control over reaction time, temperature, and concentration, as well as greater control over final nanoparticles size, shape, and homogeneity [40]. For example, Cabrera, et al. developed a latex-based microfluidic device for the synthesis of both gold and iron oxide nanoparticles without surfactants, organic solvents, or heat treatment. Both the gold and iron oxide nanoparticles could then be mixed together at varying iron oxide concentrations to produce 10 nm iron oxide NPs decorated with 4 nm gold NPs with monodisperse core sizes [41]. To support additional doping of iron NPs, Simmons et. al. used a commercially available micromixer to synthesize zinc-doped iron oxide NPs to impart greater magnetic properties for MRI [42]. The authors showed controllable zinc concentration in the final NP formulation with average < 2 nm core sizes and increased saturated magnetization. Biogenic synthesis aims to produce size and shape controlled NPs dictated by the biological processes of the organism, or biomineralization [40,43]. However, biogenic synthesis strategies still suffer from low yields.

Size, shape, and surface charge can be tuned for multiple cancer therapeutic applications, such as size-dependent hyperthermia treatment [44] and theranostics [45]. Final surface charge of synthesized nanoparticles can be used to electrostatically bind nucleic acids [46] or increase systemic circulation times [47]. Although spherical NPs are mostly used in cancer therapeutics, some research has been conducted to utilize varied shape nanoparticles such as hollow rod morphologies for drug delivery [48] and nanocube morphologies for guided chemo-photothermal therapy. Although spherical NPs are mostly used in cancer therapeutics, some research has been conducted to utilize varied shape nanoparticles such as hollow rod morphologies for drug delivery [48] and nanocube morphologies for guided chemo-photothermal therapy [38].

## 3. Magnetic Nanoparticles for Cancer Therapeutics

This section discusses the use of MNPs in chemotherapy (chemotherapeutics, biotherapeutics and radiotherapeutics), gene therapy, photothermal ablation, magnetic hyperthermia, photodynamic therapy, and direct injection of MNPs [31,32,33,49]. 

### 3.1. MNPs as Cargo Delivery Vehicle

#### 3.1.1. Drug Delivery 

Nanoparticle sized drug delivery systems have become a popular approach for drug delivery, especially in cancer therapeutics, due to their tunable physicochemical properties such as size distribution and surface modification. These parameters can be designed to impart therapeutic functionality such as conjugating various biologically active therapeutics or small molecule drugs covalently or noncovalently, increasing systemic circulation and biocompatibility, as well as employing passive and active targeting mechanisms to the tumor or therapeutic site [28]. Magnetic nanoparticles (MNPs) are a distinct class of drug delivery systems owing to their unique magnetic properties that allow for a wide range of exploitable properties such as in vivo imaging as magnetic resonance imaging (MRI) contrast agents, controlled and/or sustained drug release through magnetothermal responses, as well as aided magnetic targeting of the cargo to desired sites [29,50,51,52]. 

##### Biotherapeutics and Chemotherapeutics

Both biotherapeutics and chemotherapeutics aim to inhibit tumor growth through disruption or inhibition of cell function, such as disruption of DNA replication, protein expression, cell division processes, or anti-apoptotic mechanisms. Biotherapeutics includes the delivery of biologically active agents such as peptides, proteins [53], DNA [54], or small interfering RNA (siRNA) [55], where the delivery of DNA or siRNA can also be known as gene therapy. Chemotherapeutics, on the other hand, includes the delivery of small molecule drugs, such including paclitaxel [56] and 5-fluorouracil [57], temozolomide (TMZ) [58], doxorubicin [59]. Several researchers have recently utilized MNPs for biotherapeutic and chemotherapeutic applications [60,61,62,63,64,65,66].

**Figure 1:**
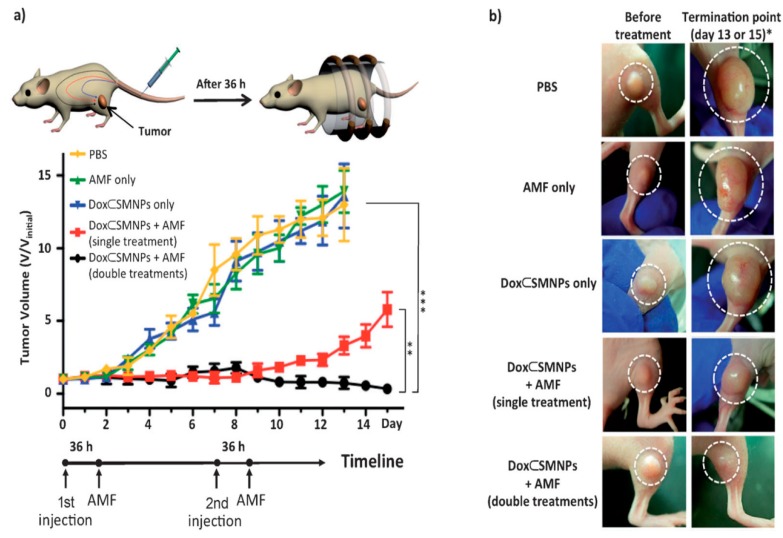
Evaluation of in vivo therapeutic efficacy. (**a**) Treatment scheme of DoxSMNPs in mouse and results of the tumor volume change over the course of the treatment (15 days) in DLD-1 xenografted mice (*n* = 3) treated with DoxSMNPs (w/and w/o application of AMF) and other controls (AMF only and PBS only). All injections were done on day 0 (and day 7 for the double injection group) when the tumor volume reached 100 mm^3^; AMF application was performed at 36 h post-injection. The best tumor suppression result was observed in the group treated with a double injection of DoxSMNPs with AMF application. The group treated with a single injection of DoxSMNPs with AMF and the other control groups (i.e., treated with DoxSMNPs only, AMF only and PBS) show either a smaller degree or none of tumor suppression effects (** *p ≤* 0.01; *** *p ≤* 0.001). (**b**) Tumor images of groups treated with DoxSMNPs w/and w/o application of AMF and other controls, before treatment (left panels) and at the termination point (right panels). * The termination point of the experiment occurred either on day 15 or when the tumor volume reached 1500 mm^3^. The figure was reproduced from [59] after permission from John Wiley and Sons.

Kievit et al. developed a chitosan-PEG-PEI coated iron oxide nanoparticle formulation functionalized with chlorotoxin (CTX) and green fluorescent protein (GFP) encoded DNA, referred to as NP:DNA-CTX [67]. Chlorotoxin is a targeting ligand specific to brain tumors, such as glioma. After administration of the NP:DNA-CTX in C6 xenograft flank tumor bearing mice, Kievit found increased NP:DNA-CTX uptake in target tumors compared to the control NP:DNA formulation, with non-significant off target cellular uptake in clearance organs such as the liver, kidney, and spleen. Kievit demonstrated the importance of copolymeric coating of iron oxide nanoparticle in order to stabilize and conjugate both targeting and gene therapy agents [68]. Wang et al. developed rod-shaped magnetic mesoporous silica nanoparticles (M-MSNs) for suicide gene therapy in which the suicide gene in situ converts the prodrug into a cytotoxic drug after cancer cell uptake [69]. Rod-like MSNs showed higher drug-loading efficiency, faster drug release, and increased gene delivery compared to spherical M-MSNs. Both nanoparticle formulations were injected into HepG2 bearing nude mice, where the MSNs were magnetically directed electromotive force (EMF) to the tumor site and/or mice underwent hyperthermia treatment using an alternating current magnetic field (ACMF). Even though individually EMF and ACMF treatments enhanced efficacy of suicide gene therapy, the combination of treatments resulted in the highest apoptotic rate of HepG2 cells and reduced tumor sizes. The evaluation of off target organs demonstrated no pathological changes suggesting little systemic toxicity. In addition, magnetic nanoparticles can be designed to control drug release in response to various internal or endogenous stimuli, including pH-, magnetic field, and hypoxia-responsive delivery [16,70,71,72]. However, the specific tumor microenvironment can be difficult to quantify dependent on cancer type and varies on a patient-to-patient basis, limiting the consistency of internal stimuli-responsive drug delivery efficacy. External or remote stimuli-based techniques can be used to further control drug delivery, such as magnetothermally triggered drug delivery. Li et al. designed a magnetothermally responsive nanocarrier/doxorubicin (MTRN/Dox) using Mn-Zn containing ferrite magnetic nanoparticles (MZF-MNPs) to form a thermosensitive copolymer coating with absorbed chemotherapeutic combined with the magnetothermal effect of MZF-MNPs to allow controlled release of the drug at the tumor site under an alternating magnetic field (AMF) [73]. The authors demonstrated magnetic targeting of MTRN/Dox increased accumulation in tumor tissues and AMF treatment was necessary for MTRN/Dox increased cytotoxicity compared to free Dox and MTRN/Dox treatment without the use of an AMF. After injection of the MTRN/Dox into nude mice bearing tumors, the MTRN/Dox with combined magnetic targeting and AMF treatment showed the greatest tumor volume reduction compared to MTRN/Dox with only magnetic targeting or AMF treatments, showing promise as liver cancer therapy. In another recent paper, Lee et al. reported a unique on-demand drug delivery using magnetothermally responsive doxorubicin-encapsulated supramolecular magnetic nanoparticles (DoxSMNPs) [59], fabricated with β-cyclodextrin (CD) motifs or adamantine (Ad) that demonstrate excellent tumor regression ability at a low dose (2.8 µg kg^−1^ Dox per injection; 1/1000^th^ of standard dose) (Figure 1).

##### Radiotherapeutics

Nanoparticles are currently being researched for their applications in the delivery of radionuclides, both α- and β- emitters, and/or radiosensitizers to induce DNA damage to tumor cells through generation of free radicals or ionic radiation [74,75,76]. Nanoparticles offer unique advantages over current radiotherapy techniques by reducing off-target tissue damage due to the non-specific nature of the treatment through passive and active targeting. Furthermore, combined therapies utilize nanoparticles for synergistic treatment, such as chemotherapy or gene therapy [77]. Munaweera et al. showed that magnetic nanoparticles containing both platinum-based chemotherapeutics and neutron-activated holmium-166 could serve as an effective chemo-radiotherapeutic for the treatment of non-small cell lung cancer [78]. Even though the study showed that the neutron-activated holmium iron garnet nanoparticles themselves did not show high cytotoxicity, the combined holmium-platinum based formulations showed a significant increase in cytotoxicity, most likely due to the fact that platinum-derived drugs also act as radiosensitizers, increasing tumor sensitivity to radiotherapy. The authors ultimately demonstrated maximized reduced tumor volumes in vivo using the proposed holmium-cisplatin nanoparticles in combination with an external alternating magnetic field to concentrate the nanoparticles. Another major hurdle of radiotherapy is the hypoxic tumor microenvironment, reducing generation of reactive oxygen species (ROS) necessary for biomolecule damage [79]. Wu et al. attempt to overcome this issue through the pro-inflammatory manipulation of myeloid derived suppressor cells (MDSCs) in gliomas, significant due to their pro-tumor production of arginases that reduce the function of adaptive immune cells [80]. The proposed modified zinc-doped iron oxide nanoparticles acted as a radiosensitizer and ROS producer while stimulating inflammatory repolarization of the MDSCs to attack tumor cells as a synergistic radio-immunotherapeutic agent for glioma treatment, as demonstrated by significantly increased median glioma-bearing mice survival rates.

### 3.2. MNPs as Intrinsic Anticancer Agents

#### 3.2.1. Cancer Immunotherapy

Cancer immunotherapy covers the range of therapies that utilize the patient’s own immune system to identify cancer cells, inhibit their proliferation, and even directly attack solid tumors. Multiple approaches have been taken to elicit immune responses for therapeutic effect, such as introducing inhibitory check point molecules (anti-CTLA-4 or anti-PD1/anti-PD-L1) [81], dendritic cell vaccines [82,83,84], adoptive cell transfer methods [85], or a combination of these approaches [86,87,88]. Combining cancer immunotherapy approaches with nanoparticles provides benefits such as a targeted delivery vehicle that can be precisely tuned to have the requisite size, shape, charge, and surface modifications to maximize delivery efficiency. Specifically, conjugation of targeting agents [11] and the use of magnetic navigation [89] can increase the localization of the therapeutic to the target site. In addition, magnetic nanoparticles can be further designed to impart additional therapeutic advantages by combining hyperthermia therapies for maximized therapeutic efficacy [90]. Many times, these nanoparticle immunotherapeutic formulations are functionalized as both a fluorescent probe and MRI contrast agent for tracking delivery in vivo^84^. In dendritic cell vaccines, nanoparticles are generally used to deliver antigens to antigen presenting dendritic cells, where the dendritic cells must then migrate to lymph nodes to activate antigen-specific cytotoxic T cells to inhibit tumor growth. Cho et al. developed a Fe_3_O_4_-ZnO core-shell nanoparticle formulation that showed efficient dendritic cell uptake without the need for additional transfection agents [83]. Furthermore, the Fe_3_O_4_-ZnO nanoparticles were observed localizing to draining lymph nodes and inducing anti-tumor immunity, as demonstrated by tenfold increased frequency of spleen CD8^+^ T cells secreting interferon gamma (IFN-γ) compared to control groups. After introduction of the labeled dendritic cells to in vivo models, the authors demonstrated significant tumor growth inhibition and increased survival rates on par with conventional protein transduction systems. More recently, nanoparticles combining check point inhibitors with either chemotherapeutic agents or cancer cell antigens have been investigated. Chiang et al. synthesized iron oxide nanoparticles with fucoidan (a polysaccharide shown to have both antitumor and immunostimulatory properties), check point inhibitor anti-PD-L1, and T-cell co-stimulatorsanti-CD3/anti-CD28 [88]. After treatment, the mice showed an increase in spleen CD8+ T cell populations, and reduced tumor associated macrophage population in the tumor environment leading to reduced Treg recruitment. Utilizing magnetic navigation to increase delivery of nanoparticles directly to the tumor site and reduce off-site effects, the authors were able to show that direct injection of their combination formulation in vivo inhibited tumor growth, reduced metastasis, and increased T cell populations required for long-term immune memory. An interesting article by Ito et al. showed the successful combined immunotherapy using interleukin-2 (IL-2) and granulocyte macrophage-colony stimulating factor (GM-CSF) with magnetic hyperthermia in mouse melanoma tumor [15].

#### 3.2.2. MNPs as Anti-Cancer Agent

MNPs have also been utilized as an anti-cancer agent by various groups. Zanganeh et al. showed cancer cell death by manipulating the iron levels by the treatment of ferumoxytol (FDA approved drug for anemia) in lung, liver and early mammary cancers [91]. Macrophages treated with ferumoxytol caused increased levels of mRNA connected with pro-inflammatory Th1-type responses. Ferumoxytol at a dose of 10 mg Fe·kg^−1^ demonstrated considerable tumor regression towards aggressive adenocarcinomas in mouse model observed by H&E staining, bioluminescence imaging, Prussian blue staining. Authors explained that ferumoxytol causes the immune cells adapting an anti-tumor ‘M1’ phenotype response, confirmed by increased presence of pro-inflammatory M1 macrophages. Moreover, the ferumoxytol treatment induced the production of ROS causing the cancer cell killing. 

### 3.3. MNPs as a Catalyst for Tumor Ablation Therapies

#### 3.3.1. Magnetic Hyperthermia 

**Figure 2:**
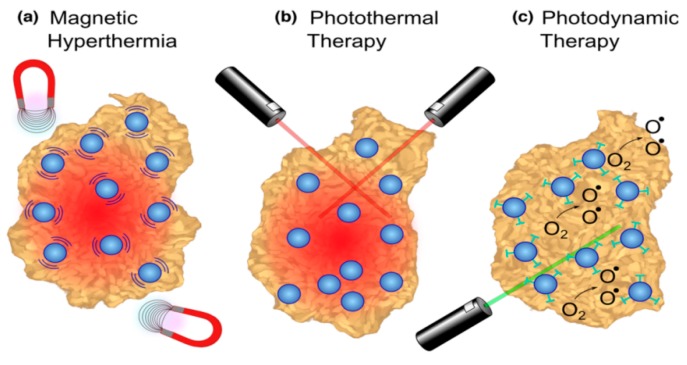
Tumor ablation therapies with iron oxide nanoparticles (NPs). (**a**) In magnetic hyperthermia, an alternating magnetic field causes iron oxide NPs to generate heat, inducing tumor necrosis. (**b**) In photothermal ablation, light absorbed by NPs is converted to thermal energy causing cell death in the vicinity. (**c**) For photodynamic therapy, photosensitizing agents attached to NPs are activated by an external light source to create singlet oxygen species that are cytotoxic to cells. The figure was reproduced from [31] after permission from Elsevier.

**Figure 3:**
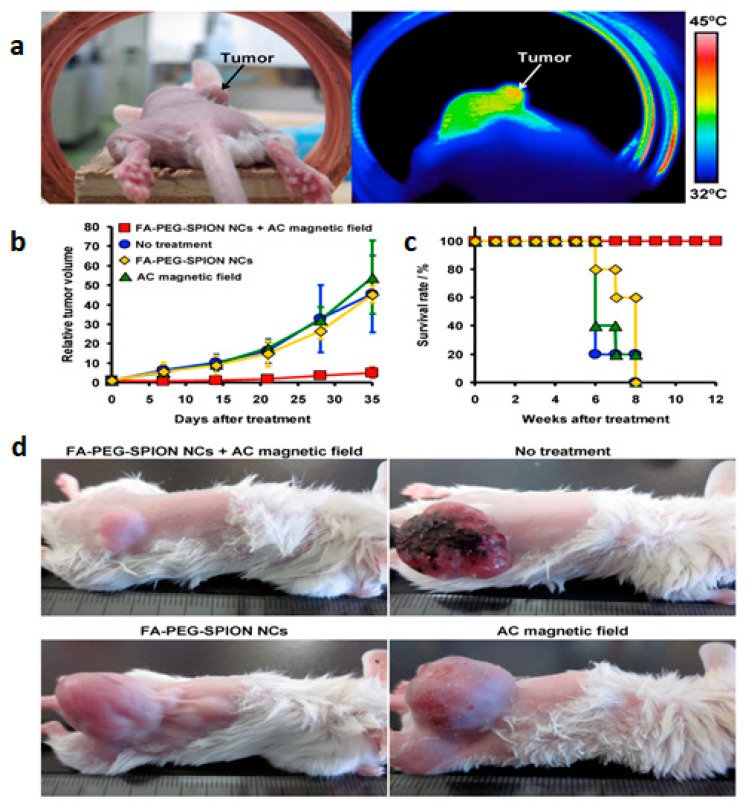
(**a**) Photograph (left) and thermal image (right) of a mouse 24 h after intravenous injection of folic acid conjugated pegylated superparamagnetic iron oxide nanoconjugates (FA-PEG-SPION NCs) under an AC magnetic field with H = 8 kA/m and f = 230 kHz. (**b**) Tumor-growth behavior and (**c**) survival period of mice without treatment and treated by intravenous injection of FA-PEG-SPION NCs, application of an alternating current (AC) magnetic field, and application of an AC magnetic field 24 h after intravenous injection of FA-PEG-SPION NCs (*n* = 5). (**d**) Photographs of mice 35 days after treatment. The figure was reproduced from [97] after permission from Ivy Spring.

Tumor ablation therapies with MNPs are generating major interest including (a) magnetic hyperthermia (necrotic tumor destruction by heat generated from MNPs upon alternating external magnetic field); (b) photothermal therapy (cancer cell death by the heat generated from MNPs upon light) and (c) photodynamic therapy (cancer cell death using cytotoxic singlet oxygen species generated from MNPs conjugated with photosensitizing agent) (Figure 2a–c) [31,92,93,94,95]. J. Kolosnjaj-Tabi, and coworkers, showed the outstanding tumor regression in mouse epidermoid carcinoma xenograft model using PEG-coated magnetite NPs after magnetic hyperthermia [96]. In another published report, Hayashi et al. demonstrated enhanced accumulation and increased magnetic relaxivity using folic acid (FA) conjugated SPIONs [97]. Moreover, the mice were placed in an external magnetic field (f = 230 kHz; Hf = 1.8 × 10^9^ A/m∙s and H = 8 kA/m) generating heat to the local tumor tissues (≈6 °C higher than surrounding tissues) causing notable tumor reduction and higher survivability (Figure 3). 

Apart from that magnetic hyperthermia is useful for controlled release of cytotoxic agents in the cancer cells using a heat-labile coating. Hu et al. recently showed the controlled release of dual drugs (Dox and paclitaxel) from heat sensitive polyvinyl alcohol (PVA) coated SPIONs using an external magnetic field [98]. Moreover, antibody conjugation with MNPs enhanced the effect of hyperthermia because of the anticancer effects of the antibody and selectivity of the cancer cells. Examples include anti-FGFR1 aptamer-tagged MNPs for enhanced magnetic hyperthermia and antibody-conjugated MNPs for enhanced anti-cancer effects of Cryptotanshinone [92,93]. Magnetic hyperthermia combined with chemotherapy demonstrated enhanced tumor regression ability. Kossatzet al. showed efficient tumor regression using combined chemotherapy and magnetic hyperthermia with superparamagnetic iron oxide nanoparticles conjugated with Nucant multivalent pseudopeptide and doxorubicin in mouse breast tumor model [94]. Another exciting report demonstrated the use of magnetic hyperthermia generated with ferucarbotran (Resovist®) to increase chemotherapeutic effects of cisplatin-induced apoptosis in human oral cancer cells in vitro [95]. For more detailed information please refer to the following article [16].

#### 3.3.2. Photothermal Ablation 

Photothermal ablation therapy uses gold coated MNPs that utilizes a NIR or visible laser light source to produce thermal heating through electromagnetic photon absorption causing cells killing (Figure 2b) [31,99]. Kirui et al. used antibody (targeting the A33 antigen) conjugated bimetallic nanoconjugates (gold shells-iron oxide core) for the targeted photothermal therapy towards colorectal cancer cells [100]. It was observed that A33 antigen expressing cells had increased NPs accumulation and resulting cell killing (~50%) when these are exposed with a NIR radiation (800 nm laser radiations at 5.1 W cm^−2^) for 6 minutes. In contrary, the non A33 expressing cells did not die under similar conditions (~5%) indicating incredible selectivity for cancer cell killing. In another published report, Larson et al. exhibited the multifunctional applications of gold-coated magnetite NPs for targeted photothermal therapy of cancer cells and multimodal imaging (MRI and optical imaging agent) [101]. 

#### 3.3.3. Photodynamic Therapy (PDT)

**Figure 4:**
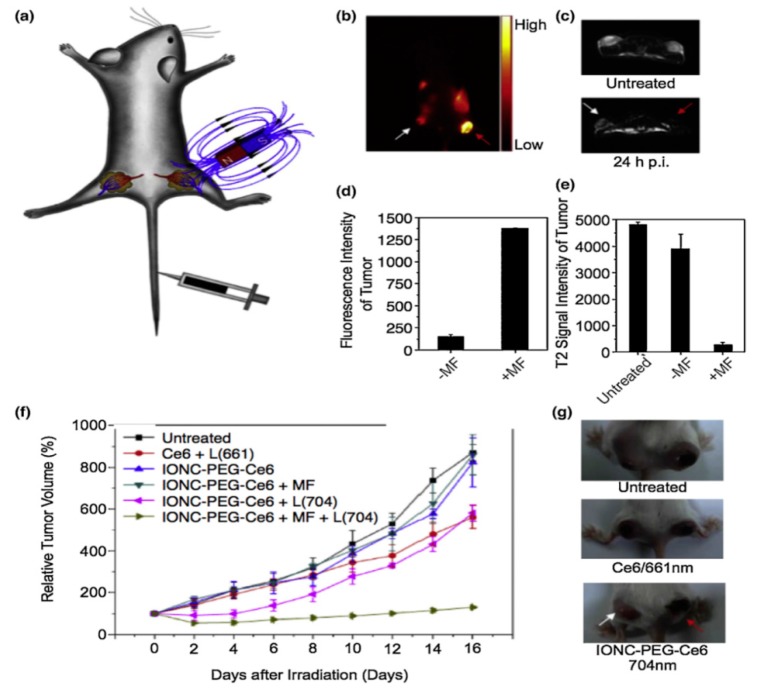
Photodynamic therapy with iron oxide nanoparticles conjugated with photosensitizing agent Ce6. (**a**) A schematic drawing to illustrate in vivo magnetic tumor targeting. (**b**) In vivo fluorescence image of a 4T1 tumor bearing mouse. (**c**) In vivo T2-weighted MR images of a mouse taken before injection (upper) and 24 h post injection (bottom). White and red arrows point to tumors without and with a magnet attached, respectively. (**d**) Ce6 fluorescence signal intensities in magnetic field (MF) targeted and non-targeted tumor regions. (**e**) T2-weighted MR signals of untreated, MF targeted and non-targeted tumors. (**f**) Tumor growth curves of different groups of tumors after various treatments indicated. Error bars were based on SD of six tumors per group. MF: magnetic field; L: light. (**g**) Representative photos of mice after various treatments. White and red arrows point to tumors without and with magnetic targeting, respectively. The figure was reproduced from [105] after permission from Elsevier.

Photodynamic therapy (PDT) utilizes a photosensitizing agent to generate cytotoxic singlet oxygen (^1^O_2_) by the excitation of an external light source ensuing free radical damage to cancer cells within a distance of 20 nm (Figure 2c) [31,102,103]. These agents are conjugated to MNPs to enhance the therapeutic efficacy. Nafiujjaman, et al. showed the PDT using pheophorbide-A (fluorescent photosensitizing agent) conjugated SPIONs upon irradiating the MNPs by a 670 nm laser source. Moreover, the nanoconjugates demonstrated profound bimodal MRI contrast/fluorescence abilities [104]. Li et al exhibited increased cellular uptake of the chlorin e6 (Ce6; photo-sensitizing agent) conjugated PEGylated SPIONs compared to pristine Ce6 [105] that were magnetically navigated to the tumor sites (Figure 4a). Figure 4b–e shows the in vivo fluorescence and MRI imaging of Ce6-conjugated SPIONs in tumor bearing mice. Additionally, considerable tumor regression was monitored upon in vivo PDT in vivo mouse model (Figure 4f–g). 

## 4. Magnetic Nanoparticles: Toxicity, Biodistribution, Pharmacokinetics

**Figure 5:**
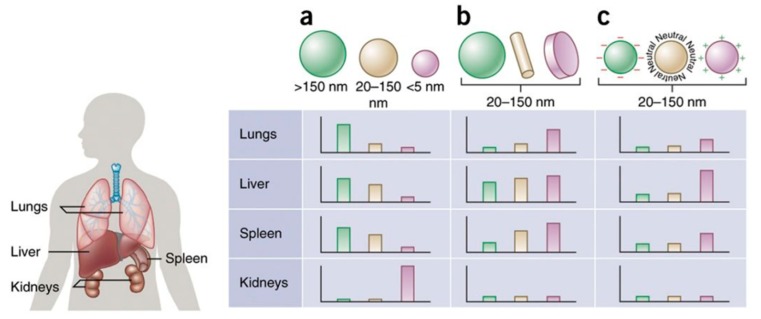
Nanoparticle size, shape and surface charge dictate biodistribution among the different organs including the lungs, liver, spleen and kidneys. (**a**) Spherical particles, including gold/magnetic nanoparticles, liposomes and polymeric micelles/NPs can vary in size and display disparate in vivo fates. Large rigid particles with diameters > 2000 nm accumulate readily within the spleen and liver, as well as in the capillaries of the lungs. Nanoparticles in the range of 100–200 nm have been shown to extravasate through vascular fenestrations of tumors (the EPR effect) and escape filtration via liver and spleen. As size increases further than 150 nm, extra NPs are captured within the liver and spleen. Small-sized NPs (<5 nm) are filtered out by the kidneys. (**b**) Novel 'top-down' and 'bottom up' fabrication tools have allowed the investigation of various geometries of NPs, including cylindrical and discoidal shapes, which have been shown to demonstrate distinct effects on pharmacokinetics and biodistribution. Various NPs shapes show exclusive flow characteristics that significantly change circulating lifetimes, cell membrane interactions and macrophage uptake, which in turn manipulate biodistribution between the different organs. (**c**) Charge of NPs stemming from distinct surface chemistries influences opsonization, circulation times, and interaction with local macrophages of organs comprising the mononuclear phagocytic system (MPS), with positively charged particles more prone to sequestration by macrophages in the lungs, liver, and spleen. Neutral and a little negatively charged NPs have longer circulation lifetimes and lower accumulation in the above mentioned organs of the MPS. In both b and c, the size of the NPs is in the range from 20–150 nm. Individual panels correspond to in vivo fates of NPs, taking into account singular design parameters of size, shape, and surface charge independent of one another, and for this reason, respective scales differ from one panel to the next. It is vital to note that in vivo biodistribution will vary based on the interaction of various these parameters. The figure and figure caption was reproduced and adapted from [110] after permission from NPG, respectively.

Toxicity, pharmacokinetics and biodistribution of magnetic NPs are crucial for their successful applications in clinics. The hydrodynamic size, surface potential, coating, interaction of NPs with reticuloendothelial system (RES) plays important role for pharmacokinetics and pharmacodynamics MNPs inside body [106]. Size also plays noteworthy role for the excretion of MNPs as small NPs can easily excrete through the renal route whereas the larger particles can be taken up by the liver and spleen before eventual degradation or excretion through the hepatobiliary route [107]. Physicochemical properties of nanomaterials such as size, structure, composition, surface charge, and surface modification contribute to the toxicity of developed magnetic nanoparticle (MNP) formulations [108,109,110]. Firstly, NPs core sizes less than 10 nm are filtered out of circulation by renal clearance and core sizes larger than 200 nm are easily sequestered by the spleen [108,111]. Next, NPs with a neutral surface charge exhibited longer circulation times compared to nanoparticles with positive or negative surface charges (Figure 5) [110]. Surface coating has demonstrated important role for the circulation of MNPs. For example, Cole, A.J. and co-workers showed that PEG-modified MNPs of 170 nm has a half-life of 12 hours [112]. Hence, it is important to carefully manipulate the circulation of the MNPs that significantly affects the biodistribution of MNPs and its biocompatibility [113] as accumulation in liver and spleen can cause off-target toxicity. Apart from that in vivo toxicity depends on the other important factors including, synthesis procedure, purity, size, surface charge, biodistribution, and pharmacokinetic properties [114].

MNPs can display toxicity by various mechanisms including, a) production of reactive oxygen species (ROS) by Fenton reaction; b) direct generation of ROS of from nanoparticle surface; c) alteration of mitochondria and other organelle functions by various affecting different cell signaling pathways. For that reason, it is crucial to assess the NPs toxicity before clinical use in cancer therapy and diagnosis [115,116,117]. Uncoated or dextran-coated superparamagnetic iron oxide nanoparticles (SPION) were shown to cause cell death in vitro [118] that could directly be attributed to generation of reactive oxygen species with the SPION as the source [119,120]. However, the extent of measured toxicity and reactive oxygen species generation was dependent on cell type [121]. Feridex, a clinically approved dextran-coated SPION formulation shown in Phase I clinical trials to be safe for patient use [122], demonstrated the limitations of data extrapolation from animal models to clinical trials.

In a biologically relevant environment, nanoparticles adsorption of plasma proteins, or opsonization, during circulation leads to the formation of a protein corona. This protein corona, which includes immunoglobulins, components of the complement system, albumins, among others, can promote receptor-mediated phagocytosis, mitigate the functionality of active targeting agents, and alter key magnetic properties such as magnetization saturation. The phagocytic uptake of these NPs by mostly resident macrophages in the liver, kidney, spleen, and lymph nodes leads to nanoparticles sequestration and clearance from blood circulation [123,124,125,126,127,128]. Moreover, due to the rapid protein adsorption on MNP surface and formation of a protein corona upon introduction to biological media, MNP toxicity in vivo is further complicated by the nano-bio interface and its biological interactions [111]. The formation of the protein corona is similarly dictated by MNP physicochemical properties, with larger hydrodynamic sizes, increased, increased surface to area volume, negative surface charge, hydrophobicity all independently showing protein adsorption [129]. While MNP surfaces can be modified to aid both passive [130] and active accumulation [131] at target sites with minimal or no systemic toxicity, considerations must be made towards acute iron overload in the localized environment causing toxic effects [132,133]. In addition, protein conformation may change upon adsorption or cause MNP aggregation triggering cellular responses with unintended, adverse outcomes [134].

Various NP surface coatings have been investigated to reduce protein adsorption and subsequently increase circulation time, the most common including polymers such as polyethylene glycol (PEG) [135,136,137], polysaccharides [138], and zwitterions [139,140]. To date, PEGylation continues to be a dominant strategy in increasing circulation times of MNPs, such as in magnetic particle imaging (MPI). In MPI, both static and alternating magnetic fields are applied to the subject such that a small volume of interest, the field-free point, containing the MNP tracer can be directly measured without any background from weakly magnetized materials [12]. Khandhar et al. tested varying loading capacities and molecular weights of PEG conjugated to poly(maleic anhydride-alt-1-octadecene) (PMAO) surface coatings of 25 nm SPIO cores. They found that 18.8% loaded, 20 kDa PEG coated SPIO tracer formulation had a 105-minute blood circulation half-life and persistent intravascular signal lasting over 3.5 hours in mice, demonstrating greatly improved blood pool imaging capabilities [141].

Recently, a novel strategy of “stealthing” MNPs from the mononuclear phagocytic system (MPS) through selective in situ adsorption of specific apolipoproteins to reduce MPS clearance in a similar manner as PEGylated NPs was shown by Magro et al [142]. The authors developed an iron oxide nanoparticles formulation with unique crystal organization suitable for specific protein docking, called surface active maghemite nanoparticles (SAMNs). The SAMNs were loaded with antibiotic (oxytetracycline) and found to forma protein corona mostly composed of apolipoprotein A1 (Apo A1) in zebrafish. The bound Apo A1 protein retained its conformational structure, suggesting that preservation of bound protein biological identity is necessary for prolonged drug delivery and avoidance of MPS clearance. Moreover, the bound Apo A1 could be used as an active targeting agent through Apo A1 transport in developing oocytes in fish ovary, evidenced by the high localization of both bare and antibiotic loaded SAMNs in zebrafish ovaries. However, more studies conducted in various animal models must be conducted to further assess the applicability of this stealthing strategy beyond fish models.

## 5. Challenges, Future Scopes, and Conclusion

Significant progress has been made with various MNP platforms toward different pre-clinical cancer theranostics applications. Despite their potential, few MNP formulations have shown success in clinical trials. Critical information still needs to be researched further to overcome complex challenges such as understanding nano-bio interactions in humans, crossing physiological and technical barriers specific to a cancer type, escaping the late endosome/lysosome system into the cytosol within tumor cells, and long-term toxicity. Nanoparticles, including magnetic NPs, face a variety of biological barriers that mitigate the localization of therapeutics at the target site, limiting the use of NPs as efficacious drug delivery vehicles and theranostics. These obstacles include NP opsonization and clearance by the MPS, nonspecific distribution, cellular internalization, endosomal escape, and drug efflux pumps. Proper surface coating strategies are required to limit the MNPs aggregation and generating ROS subsequently causing toxicity. The other challenges include tumor specific targeting to the inflamed/tumor bearing site, which will help lower dose and better efficacy of MNPs. Conjugation of anti-body, targeted agents, immunomodulatory ligands helped to increase the tumor targeting. However, less than 10% of NPs update to the tumor site makes it difficult for long, sustained therapy with low toxicity.

In addition, regulatory and industry barriers provide another set of challenges for the clinical translation of MNPs, such as adherence to good laboratory practices. A multidisciplinary approach must be taken in collaboration with regulatory institutions to evaluate the safety and efficacy of nanotechnology as a whole. MRI monitoring of nanoparticles has proved a promising aspect of MNP-based platforms in addition to recent advances in improving active targeting, controlled and sustained drug release, and synergistic multimodal therapies. Considerable contributions using mathematical modeling of multi-functional complex nanosystems, with the purpose of understanding, more specifically, the intricate interactions and efficacy, will decide whether, in the concluding analysis, a certain biomedical strategy can be effectively used. With the trend of research institutes establishing interdisciplinary nanotechnology centers and regulatory institutions developing standards specific to NP platforms, an increase in suitable pre-clinical in vivo studies can be expected to escalate to clinical trials in the coming years for the development of improved magnetic nanoplatforms for the diagnosis and treatment of cancer.

However, no single MNPs formulation has been approved for cancer therapeutic use till date. Additionally, after the withdrawal of a few MNPs based products, various regulatory safeguards have currently been initiated by the regulatory agencies, to assure a secure and efficient fundamental and translational development of MNPs. We believe the delay in successful MNPs in clinics is driven by the academic reward system, where much importance has been given to develop a novel formulations rather than focusing on the clinical translation of the accessible ones. It is important as a community to address these challenges for fast and hassle-free clinical development of these novel MNPs for cancer therapy.
